# Induction of Asthma and the Environment: What We Know and Need to Know

**DOI:** 10.1289/ehp.8376

**Published:** 2006-01-26

**Authors:** MaryJane K. Selgrade, Robert F. Lemanske, M. Ian Gilmour, Lucas M. Neas, Marsha D.W. Ward, Paul K. Henneberger, David N. Weissman, Jane A. Hoppin, Rodney R. Dietert, Peter D. Sly, Andrew M. Geller, Paul L. Enright, Gillian S. Backus, Philip A. Bromberg, Dori R. Germolec, Karin B. Yeatts

**Affiliations:** 1 National Health and Environmental Effects Research Laboratory, U.S. Environmental Protection Agency, Research Triangle Park, North Carolina, USA; 2 Division of Pediatric Allergy, Immunology, and Rheumatology, University of Wisconsin, Madison, Wisconsin, USA; 3 National Institute for Occupational Safety and Health, Morgantown, West Virginia, USA; 4 National Institute of Environmental Health Sciences, National Institutes of Health, Department of Health and Human Services, Research Triangle Park, North Carolina, USA; 5 Department of Microbiology and Immunology, College of Veterinary Medicine, Cornell University, Ithaca, New York, USA; 6 Center for Child Health Research, University of Western Australia, Perth, Australia; 7 Curriculum in Toxicology and; 8 Center for Environmental Medicine, Asthma and Lung Biology, School of Medicine, University of North Carolina, Chapel Hill, North Carolina, USA

**Keywords:** air pollution, allergy, asthma economic impact, asthma induction, asthma prevalence, biologics, indoor environment, occupational exposure, public health, susceptibility

## Abstract

The prevalence of asthma has increased dramatically over the last 25 years in the United States and in other nations as a result of ill-defined changes in living conditions in modern society. On 18 and 19 October 2004 the U.S. Environmental Protection Agency and the National Institute of Environmental Health Sciences sponsored the workshop “Environmental Influences on the Induction and Incidence of Asthma” to review current scientific evidence with respect to factors that may contribute to the induction of asthma. Participants addressed two broad questions: *a*) What does the science suggest that regulatory and public health agencies could do now to reduce the incidence of asthma? and *b*) What research is needed to improve our understanding of the factors that contribute to the induction of asthma and our ability to manage this problem? In this article (one of four articles resulting from the workshop), we briefly characterize asthma and its public health and economic impacts, and intervention strategies that have been successfully used to prevent induction of asthma in the workplace. We conclude with the findings of seven working groups that focus on ambient air, indoor pollutants (biologics), occupational exposures, early life stages, older adults, intrinsic susceptibility, and lifestyle. These groups found strong scientific support for public health efforts to limit *in utero* and postnatal exposure to cigarette smoke. However, with respect to other potential types of interventions, participants noted many scientific questions, which are summarized in this article. Research to address these questions could have a significant public health and economic impact that would be well worth the investment.

The prevalence of asthma has increased dramatically over the last 25 years in the United States and in other industrialized nations as a result of ill-defined changes in living conditions in modern Western society (Pew Environmental Health Commission 2000; Mannino et al. 2002). Exposure to air pollutants, including tobacco smoke, ozone, and diesel exhaust, increases the risk of developing asthma (Gilmour et al. 2006) and may be contributing to this trend. In addition, indoor exposures to allergens and to other biologics have been implicated in the increased incidence of asthma because more time is spent indoors and indoor environments have been made more airtight to improve energy efficiency (Bush 2001; Zeldin et al. 2006). However, other factors, including increased incidence of obesity, decreased exercise, change in diet, decreased exposure to microbial products during early life (the hygiene hypothesis), and increased viral respiratory infections (e.g., from daycare facilities), are all possible contributors to the rise in asthma incidence (Yeatts et al. 2006). Although children appear to be the population most at risk, there is growing concern that new cases are also arising in adults (Enright et al. 1999). Induction of atopic phenotype (Nguyen et al. 2003) and asthma (Gautrin et al. 2001) have both been well documented in adults in occupational settings.

On 18 and 19 October 2004 the U.S. Environmental Protection Agency (EPA) and National Institute of Environmental Health Sciences sponsored the workshop “Environmental Influences on the Induction and Incidence of Asthma.” The workshop focused on the origin of disease rather than on exacerbation of existing disease because the former is a more challenging problem and the ultimate goal is prevention. The purpose of this workshop was to review the current scientific evidence with respect to factors that may contribute to the induction (Gilmour et al. 2006; Yeatts et al. 2006; Zeldin et al. 2006) and, therefore, increased incidence of asthma. Participants addressed two broad questions: *a*) What does the science suggest that regulatory and public health agencies could do now to reduce the incidence of asthma? and *b*) What research is needed to improve our understanding of the factors that contribute to the induction of asthma and to improve our ability to manage this problem in the future? Participants broke into working groups at the end of the workshop to consider these two questions and to develop recommendations.

In this article, we provide a brief characterization of asthma and the public health and economic impacts of this disease as well as a brief description of intervention strategies used to prevent induction of asthma in occupational settings. We conclude with a summary of the findings of the workshop based on the deliberation of seven working groups. These findings are focused on ambient (outdoor) air, indoor pollutants (biologics), occupational exposures, early life stages (development), older adults and the aged, intrinsic (genetic) susceptibility, and lifestyle. The subsequent three articles in this series provide a more in-depth picture of the important contributions of allergens and other biologics (Zeldin et al. 2006), ambient air pollutants and cigarette smoke (Gilmour et al. 2006), and susceptibility factors such as age, genetics, and obesity (Yeatts et al. 2006) to the induction of asthma as well as a more detailed view of research needs and potential intervention strategies.

## What Is Asthma?

Dealing with asthma means many different things to different people. To the patient, it means episodic wheezing, coughing, and/or shortness of breath. To the parent, it may mean sleepless nights or missed workdays because of the presence of symptoms in their child. To the clinician, asthma is a complex condition that presents as multiple different phenotypes that can vary with age, gender, and race. Moreover, the frequency and severity of asthma “attacks” may have both inter- and intrapatient variability and can be triggered by diverse stimuli including aeroallergen exposure, viral infections, exercise, irritant exposure, certain medications (e.g., aspirin), and gastroesophageal reflux. To the pathologist, asthma is characterized by airway inflammation and mucus hypersecretion. To the physiologist, airflow obstruction and airway hyperresponsiveness are most relevant (Lemanske and Busse 2003).

The increases in the incidence, prevalence (incidence × duration), morbidity, and mortality from asthma during the past few decades in many parts of the world have led to renewed consideration of this disease by researchers both in basic and clinical science. A number of expert panels across several countries throughout the world, including the National Asthma Education and Prevention Program funded by the National Heart, Lung, and Blood Institute in the United States (NHLBI 1991, 1997), have been assembled to generate pathophysiological definitions of asthma and to make treatment recommendations based on disease severity [American Academy of Allergy and Immunology (AAAI) et al. 1995; Boulet et al. 1999; British Thoracic Society 1997; Eid 2004; Thole et al. 2003). The major emphasis of the 1991 NHLBI asthma guidelines (NHLBI 1991) was that asthma was an inflammatory disease; thus, while bronchospasm was clearly contributing to the clinical symptoms both acutely and chronically, research in the 1980s that included both bronchoalveolar lavage and bronchial biopsies demonstrated clearly the presence of ongoing airway inflammation. In 1997 the primary focus of the expert panels was on the early recognition and treatment of asthma to prevent or attenuate a loss of lung function over time.

Prospective birth cohort studies have revealed that for many asthmatic patients the disease has its roots in infancy (Gerritsen 2002; Taussig et al. 2003). Indeed, wheezing in children younger than 3 years has been found to be associated with the presence of inflammatory cells and mediators (Krawiec et al. 2001). Wheezing is common during infancy and early childhood because of the increased frequency of viral respiratory tract infections and small airway diameters (Cypcar et al. 1992). Exposure to cigarette smoke has also been associated with wheezing in the first year of life (Hagendorens et al. 2005). Allergen exposure in early life has been linked to allergic sensitization and later asthma, but there is little evidence for an association between early allergen exposure and wheezing in infants or children under 5 years of age (Sporik et al. 1990; Brussee et al. 2005). Distinguishing children who will be transient wheezers from those who will be persistent wheezers during later childhood has been a challenge to the clinician in terms of whom to treat, when to treat, and with what to treat during this time period (Taussig et al. 2003).

In the first decade of life, males outnumber females with asthma about 2.5:1. The sex balance becomes relatively equal during adolescence (related to remission in males and new onset in females), and in adults, female asthmatic patients tend to outnumber males (King et al. 2004). More important, however, the disease is usually more severe in adult female patients.

The majority of medications currently available to treat asthma is very useful in controlling clinical symptoms. Unfortunately, these medications merely control symptoms, they do not cure the disease. Thus, the concept of both secondary and primary prevention is now being addressed by a number of research groups.

## The Public Health and Economic Impacts

Asthma is a substantial public health burden, particularly for children, both in the number of people affected by the disease, and the related morbidity and cost. Globally, as many as 300 million people of all ages and all ethnic backgrounds suffer from asthma and the burden of this disease to governments, health care systems, families, and patients is increasing worldwide [Global Initiative for Asthma (GINA) 2004]. It is estimated that 21 million people in the United States currently have asthma, based on U.S. Centers for Disease Control and Prevention (U.S. CDC) Behavioral Risk Factor Surveillance System data (U.S. CDC 2004). Within this population 11.8 million Americans (4.2 million children under 18 years of age) had an asthma episode or attack during the same year. Fourteen million missed school days and 14.5 million missed workdays annually have been attributed to asthma (U.S. CDC 2002). Annually, nearly 1.9 million emergency department visits (U.S. CDC 2002) and 11.3 million physician office visits have been attributed to asthma (NHLBI 2004). Primary hospitalizations caused by asthma were estimated at 484,000 in 2002 (NHLBI 2004). In the same year there were 4,269 asthma-related deaths.

The economics of asthma can be divided into both direct and indirect costs. The direct costs of asthma include asthma management programs, inpatient and outpatient medical care, physician services, emergency visits, and medication. The projected cost of treating asthma in those younger than 18 years is $3.2 billion per year. Overall, the annual direct health care cost attributable to asthma is estimated to be approximately $11.5 billion. Some of the indirect costs of asthma include absence from work and school, activity limitations, sleep disturbances, and at its most extreme, death. Costs most difficult to measure are anxiety, pain, suffering, and decreased potential resulting from school absenteeism (Weiss and Sullivan 2001). Indirect costs (e.g., lost productivity) account for approximately $4.6 billion, for a total of $16.1 billion dollars (American Lung Association 2005; NHLBI 2004).

## Intervention: Lessons Learned from Occupational Asthma

An implicit assumption of this workshop was that by modifying environmental factors we could reduce the incidence of asthma. Experience with occupational asthma (OA), occurring as a consequence of sensitization to causative agents in the workplace, provides evidence that it is possible to prevent induction of asthma in some circumstances by modifying the work environment. OA accounts for about 15% of asthma in adults (Balmes et al. 2003). Because exposures in the industrial workplace are often identifiable, the offending agent can usually be isolated. Exposures can be monitored and limited, and medical monitoring can be conducted to determine the extent of the problem as well as to identify and treat new cases (i.e., secondary prevention) and document the success of preventive interventions. OA development, morbidity, and mortality have been prevented by interventions targeted to different phases of the disease process, the first two of which are germane to this workshop. Primary prevention limits exposure to causative agents and thus prevents sensitization. Secondary prevention occurs early in the disease process, preferably at a preclinical stage, to prevent progression to full-blown clinical disease. Interventions to reduce exposure of individuals to causative agents after they become sensitized or after development of mild, preclinical airways involvement constitute secondary prevention. Tertiary prevention involves treatment of established clinical disease to limit morbidity and mortality as well as limitation of exposure.

An industrial hygiene prevention hierarchy is employed in the prevention of OA. These interventions include both early disease detection and identification and reduction of workplace exposures. Where possible, substitution of agents that do not cause OA is the best alternative. Where substitution is not possible, engineering and administrative controls can be used to limit exposures. Use of personal protective equipment such as respirators is an important backup to engineering and administrative controls, but it should not be the only mode of prevention because it depends on appropriate respirator selection, fit, and function, and worker adherence to wearing respirators whenever needed. Medical screening and surveillance is used to identify affected workers after sensitization or early after development of airways manifestations but before developing full-blown OA. Both the affected worker and other exposed individuals benefit if early detection of disease leads to identification of sensitizing agents and reduction of workplace exposures. Finally, worker education is an important intervention, allowing workers to recognize and report hazardous situations. Similar exposure-reducing and education approaches have been adopted to limit exposure to sensitizing agents in other indoor environments, and some success in reducing asthma symptoms in young children has been demonstrated using these approaches (Zeldin et al. 2006).

OA has been prevented successfully in several settings. An impressive example was elimination of asthma outbreaks in the enzyme detergent industry in the late 1960s and early 1970s (Cathcart et al. 1997; Schweigert et al. 2000). In this instance a threshold level was established below which allergic sensitization is rare and OA does not occur. A more recent example has been marked reduction in OA caused by the use of powdered natural rubber latex gloves in the health care industry (Allmers et al. 2002). Although the scope of prevention strategies used in the occupational setting may be very narrow relative to that which may be needed in the population as a whole, successes in this arena do suggest that, given enough information about the underlying causes and logistically feasible intervention strategies, induction of asthma can be prevented, at least in adults.

## Workshop Findings: Public Health Measures and Research Needs

Following presentations that have been captured in this and the three articles that follow, workshop participants divided into seven topic-based discussion groups. Each group included researchers, regulators, and individuals involved in a variety of public health activities. The charge to the breakout groups was to consider in the context of their particular topic area the two broad workshop questions: *a*) What does the science suggest that regulatory and public health agencies could do now to reduce the incidence of asthma? and *b*) What research is needed to improve our understanding of the factors that contribute to the induction of asthma and to improve our ability to manage this problem in the future? Having considered these questions, the groups developed the following set of workshop conclusions:

### Ambient air pollutants.

There is sufficient epidemiologic and animal data to suggest that some synergism exists between exposure to air pollutants (primarily outdoor) and biologics (primarily indoor) in the induction of asthma in children and possibly adults (Gilmour et al. 2006). However, a number of questions need to be answered in order to make appropriate regulatory decisions for ambient air. A coordinated research effort that includes epidemiology, clinical, and animal studies is needed to address the following research questions:

- Which air pollutants are of greatest concern and at what concentrations? Is the magnitude of the inflammatory response (or some other biomarker) predictive of this effect? What does the dose–response curve look like? Is there a threshold? What are the consequences of acute vs chronic exposure? Are there indirect effects of air pollutants (e.g., climate change) that could contribute to the problem?
- What is the relative contribution of different air pollutants to the induction of disease? Is there synergy (or antagonism) among different air pollutants or with other characteristics of western lifestyle (e.g., hygiene, viral infections, diet)?
- What is the basis of genetic susceptibility, and has that changed as a result of gene–environment interaction over the past 20 years in a way that is associated with susceptibility to air pollutant exposures? Are there particular periods of life when exposures to air pollutants are more critical (windows of vulnerability)?

### Indoor air.

The discussion of indoor air pollutants focused on biologics (mold, dust mite, cockroach and rodent infestations), dampness, and environmental tobacco smoke (ETS). As noted earlier, exposure to ETS plays a role in the development of asthma. Public health measures to limit exposure to ETS should reduce the incidence of the disease (Gilmour et al. 2006; Yeatts et al. 2006). The potential benefits of reducing exposure to allergens in order to prevent induction of asthma are less clear. The National Academy of Sciences (2000) reached a similar conclusion. Current data indicate that reducing exposure will reduce morbidity among sensitized children who already have asthma but fall short of linking reduced exposure with a reduction in incident asthma. Cleaning up and preventing mold, dust mite, and cockroach and rodent infestations as well as controlling dampness will reduce allergens in indoor environments. However, as noted below, additional research is needed to develop more effective cleanup procedures. Education of the public and building inspectors will promote identification and clean up of sources of allergens. These important first steps should be taken now. At this time, the development of regulations to assure home and school environments that are less likely to contribute to the development of asthma is precluded by the lack of research supporting specific interventions. Research is needed to address the following questions:

- What are the important sources of allergens? What levels of allergen exposure constitute a risk for the development of asthma? What is the relative contribution of different agents to the development of asthma, and what medical/health benefits are achieved when remediation occurs?
- What precautions during the construction and maintenance of buildings can reduce indoor exposures to agents likely to be involved in the development of asthma? What are the most effective cleanup measures for contaminated buildings?
- Do previously unrecognized agents (e.g., microbial volatile organic compounds, adjuvant activity, toxins, and/or proteases) pose a risk with respect to asthma?
- How can (standardized) approaches be developed for measuring exposures and outcomes in epidemiologic studies? What are the best approaches for collecting pollutants and allergens from different surfaces?
- Is IgE the most appropriate biomarker of effect? What is the role of non-IgE–mediated pathways in the development of asthma, and does this role suggest other biomarkers of effect that should be considered?

### Occupational.

As noted earlier the occupational setting is more controlled than most other settings (e.g., homes, general outdoor environment). Where data are sufficient, safe exposure standards should be set (e.g., wheat allergen) and maintained. However, there are approximately 300 occupational asthmagens. For most of these there are not sufficient data to set standards nor is it practical to set standards for each one. A more general approach includes reducing exposures to known sensitizers to the lowest levels possible, implementing medical monitoring for workers exposed to asthma-inducing agents, and improving the education (regarding work-related asthma) of exposed workers, their employers, and the physicians who treat them. Companies that produce and/or sell products that contain known asthma agents should implement product stewardship programs to educate workers in both the parent plants and downstream user plants. One goal of future research should be to develop methods to identify potential asthmagens before they are introduced into the workplace. Once an asthmagen is recognized, issues with respect to exposure, dose response, and route of exposure need to be resolved in order to implement appropriate controls. The occupational setting provides an excellent opportunity to maximize the benefit of coordinated human and animal studies to address important research questions such as:

- What are the mechanisms associated with occupationally induced asthma, particularly in association with low-molecular-weight agents (haptens)? (See Yeatts et al. 2006 for distinction between proteins and haptens in OA.) Can we develop mechanistically based tests and/or structure/activity relationships that would identify the potential to induce asthma before a chemical is introduced into the workplace? What is the role of IgE versus other mechanisms that might contribute to induction of asthma by low molecular weight chemicals?
- What are the most appropriate animal models for asthma, especially when studying low-molecular-weight agents?
- What are the effects of exposures to combinations of chemicals? How do irritants and sensitizers interact? How do non-work-related exposure and lifestyle affect the development of work-related asthma?
- What are the most relevant exposure scenarios? What are the best strategies for assessing peak exposures? What are the most relevant routes of exposure? When there are multiple routes of exposure, how does that impact the induction of disease?
- What can we learn about genetic susceptibility and asthma in older adults by studying occupational settings? Are certain groups more at risk of developing occupational asthma, for example, women and certain ethnic/racial groups, or at higher risk for asthma in general?
- What are the biomarkers that would be most appropriate both for animal studies and medical monitoring? What is the best approach to implementing medical monitoring in the workplace and using the results to guide preventive interventions? How can we maximize participation in medical monitoring programs?
- What is the best way to demonstrate successful interventions? Which interventions are the most effective in preventing occupational asthma?

### Early life.

Young children are more susceptible than adults to induction of asthma by environmental factors. The sources of this disparity include different exposure and dose to tissue characteristics and both qualitative and quantitative differences in the respiratory, immune, endocrine, and nervous systems during stages of rapid growth and development. There is much we do not understand about the risks during the period from gestation through the first year of life. One thing we do understand is that maternal smoking and exposure to ETS during this period increases the risk of developing asthma and that smoking even before the onset of pregnancy should be considered a risk (Gilmour et al. 2006; Yeatts et al. 2006). Therefore, public health measures should include regulations and education designed to prevent these exposures. The impact of other types of exposures is less clear. In particular, conflicting data on the effects of early life exposures to allergens and bacteria make it difficult to articulate effective public health strategies with respect to such exposures. Viral lower respiratory infections during early infancy, especially those associated with wheezing, are a risk factor, but reliable methods of preventing these are not available, and again the data are inconsistent with some studies suggesting that viral infections have beneficial effects. Also, little is known about the impact of most medications or current vaccination strategies on the development of asthma.

More research is needed to provide public health officials with the information they need to ensure that *in utero*, home, school, and day care environments do not contribute to the induction of asthma during the early stages of development. Investigators should target children specifically for research at these early stages rather than trying to extrapolate from them at later stages in life. Efforts should be made to increase expertise in the area of developmental biology. Epidemiology and clinical and animal studies are needed to address the following questions:

- How do the growth and development of respiratory, immune, endocrine, and nervous systems during gestation and the first year of life relate to the risks of developing asthma? What are the critical milestones in development of these systems? Can we identify major windows of vulnerability to environmental exposures?
- What biomarkers can be identified for evaluating interventions?
- What are the at-risk phenotypes and genotypes?
- Are there gender differences?
- Do current data support the hygiene hypothesis? Is exposure to some bacteria (e.g., gram-negative bacteria containing endotoxin) or other infectious agents during early life beneficial? If so, what kind, how much, and when?
- What is the effect of exposure to allergens during gestation and during the first year of life?

### Older adults.

Asthma incidence and prevalence is just as common in older adults as in younger adults. Although the type of airway inflammation of asthma differs from that of chronic obstructive pulmonary disease (COPD), measures to limit the respiratory effects of environmental exposures apply equally to patients with asthma and COPD. Regulations and education that minimize exposure of older adults to ETS, criteria air pollutants (ozone, sulfur oxides, nitrogen oxides, particulate matter), allergens, and volatile organics are warranted because their respiratory reserve is less than at younger ages. Because viral infections in this age group pose significant health problems that are compounded by asthma and other types of chronic respiratory disease, vaccinations for influenza are important as well as simple hygiene measures such as hand washing. Older adults deserve more attention from the asthma research community.

Research needs identified by the ambient and indoor air breakout groups are also important for older adults, so this group should be included in the design of those studies. In addition, the following research questions are unique to this group:

- What specific environmental factors and individual characteristics increase the risk of developing new disease in older adults (as opposed to exacerbation of existing asthma)? Do these risk factors differ from those for children?
- What is the natural history of asthma in older adults?
- What methods will effectively improve the ability of primary care physicians to diagnose asthma in older adults?
- Because mortality is a serious problem in this population, and comorbidity is common, what are the risk factors that influence asthma progressing to death in this group?

### Intrinsic (genetic) susceptibility.

Considerable evidence from family-based studies and animal models supports a role for genetic background in the pathogenesis of asthma. A better understanding of genetic susceptibility and interaction with environmental factors is necessary for development of intervention strategies and a means to identify at-risk individuals.

A number of new technologies (e.g., genome sequencing and genetic engineering) are now available to investigate the genetic basis of complex diseases such as asthma. These tools provide new opportunities for research that should ultimately improve the effectiveness of our public health policies and research design. Because asthma is a polygenic disease with important interactions with environmental exposures, it is necessary to think in a less reductionistic manner and to emphasize interaction and communication between epidemiologists, clinicians, and basic scientists. Epidemiology and genomewide scans should be used in a complementary way. Furthermore, animal and human studies should be complementary, with each providing hypotheses for teting the other. These approaches could help us to answer the following research questions:

- What is the genetic basis of asthma sub-phenotypes?
- Are different genes important at different stages of life, for different types and levels of exposure?
- What is the impact of environment on genetic predisposition to asthma (exacerbation and increased incidence)?

Use of genetic information by regulatory and public health agencies should be considered in strategies designed to reduce the incidence of asthma in at-risk populations. Currently, the higher risk of asthma in certain ethnic and socioeconomic groups has raised the issue of environmental justice in the regulatory arena. Ethical, economic, and legal considerations include the following:

- How should genetic counseling be applied to complex diseases such as asthma?
- What is the most effective way to communicate between scientists and the public about genetic information without raising concern? Can genetic information be used to develop broader public health awareness?

### Lifestyle.

Because the increased incidence of asthma seems to be due to some aspect of “Western” lifestyle, the workshop included a discussion of how changes in lifestyle apart from, or in concert with, changes in environmental exposures might influence the induction and incidence of asthma. Again, ample evidence that smoking contributes to the induction of asthma was noted, and public health actions to discourage smoking, particularly in women of childbearing age and in the presence of young children, were recommended. Smoking may also contribute to irreversible airway obstruction in persons with asthma, thereby further reinforcing the need to discourage smoking. Rapid urbanization and its associated “Westernized” lifestyle have led to decreased activity levels, increased consumption of processed foods, and increased obesity in the population. All these factors may contribute singly or in concert to asthma onset although the relationships need to be better elucidated. Clearly, interdisciplinary research with respect to other lifestyle issues is needed to provide the science necessary for appropriate public education.

Apparent links between the increased incidence of asthma and obesity described at this meeting as well as the potential for other lifestyle influences suggested several research directions at both the individual and population levels:

- Are there common mechanisms and/or causes for the increase in obesity and asthma (especially in women)?
- How do diet and exercise affect the development of asthma? Is there a role for anti-oxidants, omega-3 fatty acids, nonrefined carbohydrates, or other additives and nutrients in the prevention of asthma?
- What is the influence of breast milk and infant diet on the development of asthma in children? Is there a critical period for breast feeding? Is maternal diet important during gestation and breast feeding? Is the mother’s allergy/asthma status important?
- What is the best way to promote healthy lifestyles (behavior modification)?
- How does rapid urbanization contribute to asthma incidence?
- Does stress (not discussed at this meeting but an issue nonetheless) contribute to the induction of asthma?

## Summary

In the past few decades, increases in the incidence and prevalence of asthma worldwide have resulted in increased morbidity and mortality and have sparked renewed interest in both basic and clinical research related to this disease. Much of the newly acquired information derived from this research was reviewed during the workshop “Environmental Influences on the Induction and Incidence of Asthma*”* and this information is summarized in this and the three subsequent articles in this mini-monograph. In discussing potential actions that public health agencies could take based on currently available science, there is strong scientific support for efforts to limit *in utero* exposure to cigarette smoke. However, with respect to other potential types of interventions, participants noted many more questions than answers. A number of scientific questions were clearly articulated, and it is apparent that public health agencies are eager to have the answer to these questions. Research to address these questions could have a significant public health and economic impact that would be well worth the investment.
